# Rich-Club Phenomenon in the Interactome of *P. falciparum*—Artifact or Signature of a Parasitic Life Style?

**DOI:** 10.1371/journal.pone.0000335

**Published:** 2007-03-28

**Authors:** Stefan Wuchty

**Affiliations:** Northwestern Institute on Complexity, Northwestern University, Evanston, Illinois, United States of America; Institute for Genomic Research, United States of America

## Abstract

Recent advances have provided a first experimental protein interaction map of the human malaria parasite *P. falciparum*, which appears to be remotely related to interactomes of other eukaryotes. Here, we present a comparative topological analysis of this experimentally determined web with a network of conserved interactions between proteins in *S. cerevisiae, C. elegans* and *D. melanogaster* that have an ortholog in *Plasmodium*. Focusing on experimental interactions, we find a significant presence of a “rich-club,” a topological characteristic that features an “oligarchy” of highly connected proteins being intertwined with one another. In complete contrast, the network of interologs and particularly the web of evolutionary-conserved interactions in *P. falciparum* lack this feature. This observation prompts the question of whether this result points to a topological signature of the parasite's biology, since experimentally obtained interactions widely cover parasite-specific functions. Significantly, hub proteins that appear in such an oligarchy revolve around invasion functions, shaping an island of parasite-specific activities in a sea of evolutionary inherited interactions. This presence of a biologically unprecedented network feature in the human malaria parasite might be an artifact of the quality and the methods to obtain interaction data in this organism. Yet, the observation that rich-club proteins have distinctive and statistically significant functions that revolve around parasite-specific activities point to a topological signature of a parasitic life style.

## Introduction

The application of an adapted Yeast-two-hybrid technique allowed for the determination of a core set of protein interaction in the human malaria parasite *P. falciparum*, roughly covering 25% of the predicted proteome [1]. Statistical analysis of the network structure resulted in the presence of scale-free behavior. This well established inhomogeneity of biological networks highlights the role of hubs, a small minority of strongly connected proteins [2], [3], which govern a networks integrity. Another special, yet general feature of biological networks is their tendency to shape densely connected and well pronounced modules that largely share similar functions. An evolutionary corollary to modularity comes from the observation, that tightly connected modules not only show a high degree of functional homogeneity but are largely evolutionary conserved as orthologs in other organisms [4], [5], [6]. In particular, a network comparison of the currently available interactome of *P. falciparum* revealed that functional modules which are largely conserved in the comparative set of other eukaryotic organisms are rudimentary present in the parasites interactome [7].

The modern picture of networks as being governed by highly connected nodes connecting and/or maintaining the integrity of a single module [8] has been recently nurtured, yet challenged by the determination of the so-called ‘rich-club’ phenomenon [9]. This property refers to the tendency of hubs to be well connected to each other. Essentially, highly interacting nodes are more likely to form tight and intertwined sub-graphs (clubs) than their less connected counterparts. In a social context, a strong rich-club phenomenon indicates the dominance of an “oligarchy” of highly connected and mutually communicating individuals. In biological systems, many well defined functional sub-communities are governed and loosely connected by highly interacting proteins [8]; however, such hubs lack strong inter-connectivity among each other. Protein-protein interaction networks, however, do not show rich-club behavior [9]. In fact, protein hubs appear to be organized in remotely placed, yet functionally homogeneous sub-communities and are largely linked to less well conjoined proteins.

In the light of these results, we present an analysis of the experimentally determined interactome of *P. falciparum*, pointing to the surprising result that the underlying network features an ‘oligarchy’ of highly interacting proteins. In total contrast, we find that evolutionary conserved interactions between *Plasmodium* proteins that we derived from *S. cerevisiae, C. elegans* and *D. melanogaster* preserve the absence of any oligarchy of dominating hubs. Combining the interactions sets from different origin, we observe not only a minimal overlap, but also see that interactions appear to be downright spatially separated from each other. Highlighting the network vicinity around the oligarchy of proteins in the experimentally obtained interactions, we find that the rich club revolves around parasite specific functions and is embedded in a sea of interologs. In particular, we find an enrichment of secreted proteins while orthologous proteins are considerably diluted. This particular observations as quantified by the detection of the rich club nodes which appear to be parasite specific leaves us with questions about the parasite in general and the current interaction data of its proteins in particular.

## Methods

### Determination of Rich Club Phenomenon:

The so-called rich-club phenomenon is quantitatively defined by the rich-club coefficient *Φ(k)* [9]. Denoting by *E_k_* the number of edges among the *N_≥k_* nodes which have more than *k* interaction partners, the rich-club coefficient is expressed as
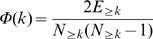
where *N_≥k_*(*N_≥k_*−1)/2 represents the maximally possible number of edges among *N_≥k_* nodes. An appropriate choice for the normalization of the rich-club coefficient is provided by the ratio
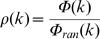
where Φ*_ran_*(*k*) is the rich-club coefficient of a random network with the same degree distribution *P(k)*. The choice of pairs of links, whose end nodes are exchanged, allows us to obtain a maximally random network while the degree distribution is preserved [9], [10]. In order to have a reasonably large ensemble, we repeat the randomization process *10,000* times. Binning nodes according to their degrees k we obtain a degree dependent mean value of the rich-club coefficient by averaging over all *ρ*'s in each bin. A ratio larger than one, *ρ (k)>1*, is the actual evidence for the presence of a rich-club phenomenon, leading to an increase in the inter-connectivity of large degree nodes in a more pronounced way than in the random case. This process is well displayed by the presence of an oligarchy of highly interacting nodes that are well connected among each other. On the contrary, a ratio *ρ (k)<1* points to a lack of inter-connectivity among large degree nodes which are separated in distinguishable modules.

### Protein Interaction Data:

The application of an adapted Yeast-two-hybrid technique allowed for the determination of a core set of *2, 811* interactions among *1, 308* proteins in the human malaria parasite *P. falciparum* [1]. As for yeast and worm specific protein interaction data, we utilized the DIP database [11], providing *17, 346* interactions among *4, 928* proteins of *S. cerevisiae* and *3, 926* interactions among *2, 718* proteins of *C. elegans*. As a reliable source of interactions among proteins of *D. melanogaster*, we utilized *6, 222* proteins and *16, 914* links [12] from a two-hybrid study.

### Orthologous Protein Data:

As a source of reliable orthologous protein information we utilized the InParanoid database [13]. The algorithm for detecting orthologous relationships is based on pairwise similarity scores which are by default calculated with BLASTP. Mutually best hits between two sequences from different species serve as main orthologs that form an orthologous group. Other sequences are added to this group if they are closely related to one of the main orthologs and are known as “in-paralogs.” A confidence value for each group member is provided by a standard bootstrap procedure and shows how closely related a protein sequence is to the main ortholog. Because of the difficulties in the detection of similarities arising from the parasites specific genome composition [14], we only selected the main sequence pairs of each orthologous group allowing us to obtain *1, 024* proteins in S. cerevisae with putative orthologs in *P. falciparum.* Analogously, we obtain *1, 333* orthologous pairs in *C. elegans* and *1, 351* in *D. melanogaster*.

### Secretome:

To establish infection in the host, malaria parasites export remodeling and virulence proteins into the erythrocyte. Recent studies independently uncovered a host cell targeting (HCT) signal that allows proteins to cross into the human erythrocyte cell by passing several membranes [15], [16], [17]. Combining these data sets, we compile a list of *525* proteins in *P. falciparum*.

### Enrichment:

According to their placement in layers around the rich-club proteins, we pool proteins or interactions in groups from the rich-club (layer #0) up to a layer #*l*, in question. In each group #*l*, we determine the fraction of proteins or links 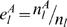, that have a certain feature *A*, where *n^A^_l_* is the respective number of proteins or interactions with feature *A* and *n_l_* is the total number of proteins or interaction in group *l*. As an appropriate null-model, we randomly distribute feature *A* among proteins or interactions, and analogously determine their respective fraction in group *l*, 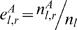. Thus, for each group *l* we define the enrichment of a feature *A* as 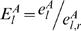, which has the *l*-independent value *E_l_ = 1* if the underlying distribution of *A* was random. According to the definition of groups *E_l_* converges to 1 while reaching the outer layers. In order to have a reasonably large ensemble, we repeat the randomization process *1,000* times and average over all *E*'s in each group. An enrichment of larger than one, *E^A^_l_*>1, is a signal for a significant over-representation of feature *A* in group *l* and *vice versa*.

## Results

Quantitatively, the rich-club phenomenon is defined by the normalized rich-club coefficient *ρ (k)*. Comparing fractions of actually existing links between nodes that have at least *k* interaction partners with the maximally possible number of edges in both the underlying and randomized networks *ρ* allows the determination of significant network patterns. In particular, a ratio larger than one, *ρ*>1, points to the presence of a rich-club, reflecting an increase in the inter-connectivity of hubs and *vice versa* [9].

**Figure 1:**
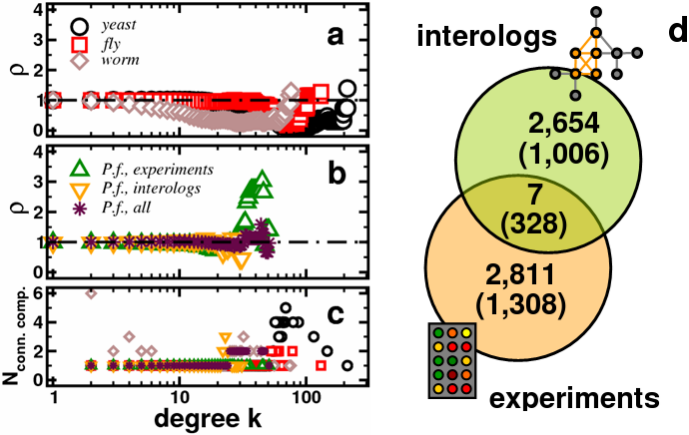
Determination and features of rich-clubs. (a) The distributions of the mean rich-club coefficient *ρ* in protein interaction networks of *S. cerevisiae, D. melanogaster* and *C. elegans* run through significant minimas, *ρ (k)<1*, indicating the absence of any rich club phenomenon. In particular, the corresponding distributions reach a minimum at *k _yeast,fly_ = 70* and *k _worm_ = 58*. (b) Focusing on a network of experimentally determined protein interactions in the human malaria parasite *P. falciparum*, we obtain a fundamentally different picture. A considerable increase of *ρ* at elevated levels of connectivity peaking at *k = 45* suggests the presence of an oligarchy of highly interacting and well intertwined proteins. We also observe a minimum at *k = 19*. Assuming that interactions between proteins in the eukaryotic networks in (a) that have an ortholog in *Plasmodium* are conserved as well, we observe that the network of interologs lacks this feature. Combining both interaction sets, the rich-club signal we encountered in the experimental interaction network weakly prevails at the same degree cut-off. (c) Since the absence of any rich-club phenomenon points to the presence of well defined sub-networks, we calculate the number of disconnected sub-networks emerging from interactions between proteins with increasing degree cut-offs. We observe that the sub-networks of yeast, fly and worm expectedly break into parts at cut-offs *k* where *ρ (k)<1*. Noteworthy, we obtain the largest number of disconnected components at degree cut-offs, where the corresponding distributions of *ρ* reach a minimum. In turn, sub-networks of experimentally determined protein interactions in *P. falciparum* keep their integrity. Inheriting this feature from its model organisms, evolutionary conserved protein interactions not only break into two disconnected parts but also keep this characteristic upon combination with experimental interactions in *Plasmodium*. (d) Although similar in their sizes, interactions in both networks only overlap to a very small but significant extent (*P<10^−4^* by assuming a hypergeometric distribution), while a considerable and significant amount of proteins (in parentheses) appear in both the experimental and evolutionary conserved protein interactions network (*P<10^−4^*).

As representative examples of well established and investigated protein interaction networks, we analyzed a network of *17, 346* interactions among *4, 928* proteins of *S. cerevisiae* and observe a distribution of *ρ* reaching a minimum at k = 70, a strong signal that points to the absence of any rich clubs (Fig. 1a). Analogously, we find similar minima around *k = 58* for the web of *3, 926* interactions among *2, 718* proteins in *C. elegans* and *k = 70* for *16, 914* interactions between *6, 222* proteins in *D. melanogaster*. In order to verify the putative presence of remotely placed modules we extract sub-networks of proteins that have a degree ≥*k* in the underlying interactomes. Indeed, we find that sub-networks composed of nodes that have at least the number of interactions where minimas occur in the distributions of *ρ (k = 70)*, break into the largest amount of disconnected parts, validating our assumption that the underlying organism-specific networks indeed are composed of a small number of remotely placed sub-communities (Fig. 1b). Changing our focus on a core set of *2, 811* experimentally determined interactions among *1, 308* proteins in *P. falciparum* [1], we observe that this network shows a slightly mixed rich-club signal encountering a minimum at *k = 19* and reaching a maximum at *k = 45* (Fig. 1b). Noteworthy, such a signature has been observed in pure scale-free networks [9], that lack any clustering and degree correlations. The observation that sub-networks where each protein has at least a certain degree in the underlying full web are organized in one connected component validates our assumption that the underlying network indeed features an oligarchy of highly connected proteins (Fig. 1c). Even at the minimum in the distribution of the rich-club coefficient *ρ* at *k*≥19 the network keeps its integrity.

In the light of this anomaly, we wonder if this effect is limited to the experimentally obtained interactions only, or prevails in evolutionary conserved links that we obtain from interologs in the eukaryotic interactomes. Utilizing orthologous protein information from the InParanoid database [13], we obtain a network of *1,006* proteins embedded in *2, 654* conserved interactions in *Plasmodium* that we derived from interactions in *S. cerevisiae, C. elegans* and *D. melanogaster*. Comparing the experimentally and evolutionary derived sets of interactions, we observe a very small but statistically significant overlap of links (Fig. 1d). In the distribution of the normalized rich-club coefficient of conserved protein interactions we find (Fig. 1b) a minimum at *k*≥21, pointing to the absence of any rich club phenomenon, supported by the observations that sub-networks of different degree cut-offs split into disconnected parts (Fig. 1c).

However, combining the two data sets in a network of *5,458* interactions between *1, 986* proteins and repeating our analysis we observe that the initially found mixed rich-club signal weakly prevails (All protein interactions can be found in Table S1).

**Figure 2:**
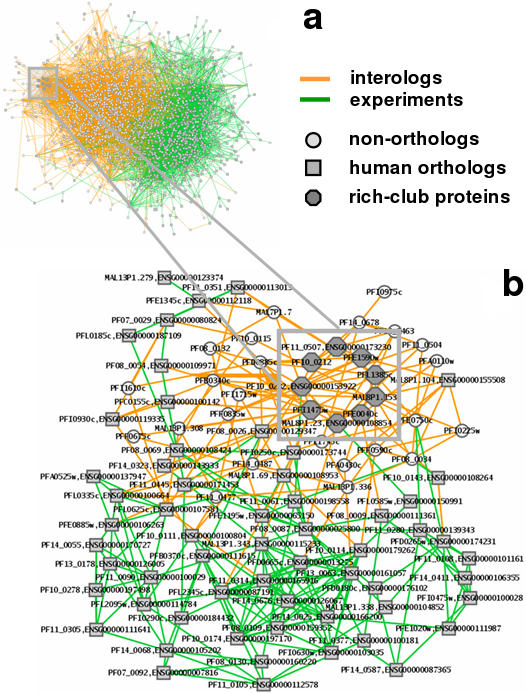
Structure of the rich-club. (a) Combining experimentally and evolutionary conserved interactions in *P. falciparum* we obtain a network of *5, 458* interactions among *1, 986* proteins. Recalling that overlaps between these two data sets is minimal we obtain a visually striking image by coloring interactions according to their origin, allowing us to observe that both types of interactions appear to be largely separated. (b) Here, we focus on the sub-network that is spanned by proteins that have at least *19* neighbors, a degree cut-off that corresponds to the minimum of the rich-club coefficient in the network of experimentally determined interactions. Confirming our previous observation, we find a strong separation of areas that largely correspond to either experimental or evolutionary interactions. Experimentally determined interactions appear to embed proteins that are part of the rich club (inset). Notably, in the immediate vicinity of the oligarchy of nodes that are strongly connected among each other, we find a relatively small number of proteins in *Plasmodium* that have a human ortholog. Clarifying, we labeled each protein of *Plasmodium* with its human ortholog. However, in a small number of steps away from the rich-club, we encounter a lot of proteins with human orthologs, which are largely organized in evolutionary conserved interactions.

As for a qualitative inspection of these networks we show a graphical depiction in Fig. 2a. Coloring each interaction by its origin, we observe that the different data sets are almost spatially separated from each other when applying a standard graph layout algorithm provided by the Cytoscape program [18]. In Fig. 2b, we focus on the sub-network that is spanned by proteins that have at least *19* neighbors, a degree cut-off that corresponds to the minimum of the rich-club coefficient in the network of experimentally determined interactions. Confirming our previous observation, we find a significant separation of areas that largely correspond to either experimental or evolutionary conserved interactions. Experimentally determined interactions appear to embed proteins that are part of the rich club (inset), mostly carrying parasite specific functions. In particular, the *9* proteins being embedded in an oligarchy of highly connected nodes proteins are predominately important for the invasion of the human host in the merozoite stage [1], [19], [20], [21], [22], where the surface proteins PFL1385c, PFI1475w and PFE0040c play a pivotal role. Especially the latter protein causes attention since it is the only one in the rich club that carries an export signal, allowing it to enter the human host cell [15], [16], [17]. Utilizing human orthologs as a common denominator, we find that one third of the rich club proteins have a human ortholog. Clarifying, we labeled each protein of *Plasmodium* with its human ortholog in Fig. 2b, allowing us to see that a relatively small number of proteins in *Plasmodium* have a human ortholog in the immediate vicinity of the rich-club proteins. However, in a small number of steps away from the rich-club, we encounter a lot of proteins with human orthologs, which are largely organized in evolutionary conserved interactions.

**Figure 3:**
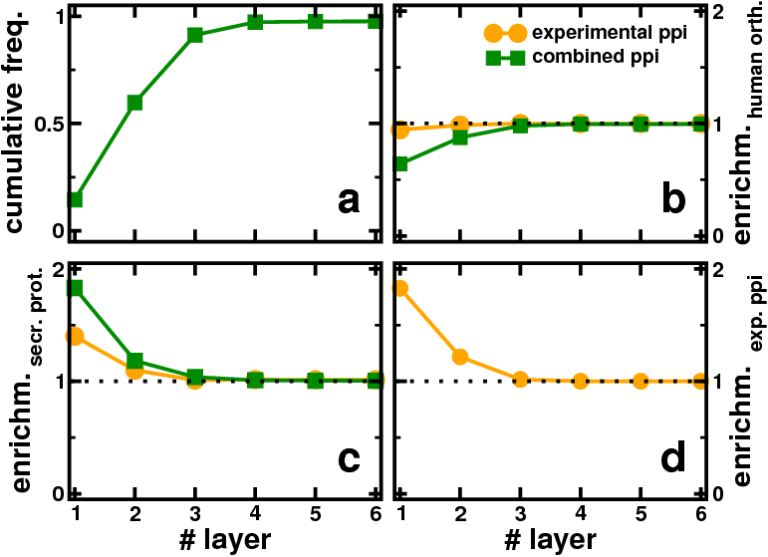
Statistics of layers around the rich-club. (a) Here, we show the cumulative frequency distribution of the layers around the rich-club (layer #0) toward the periphery of the network (layer #6). In particular, layer *#n* refers to all the nodes that can be reached *n* steps away from the rich club of proteins. Reaching the second layer we already accumulate half of all the proteins in the network. These growing pools of proteins obtained by consecutive layers are the references for the following observations. (b) Analyzing the composition of consecutive layers we find no significant enrichment of human orthologs in experimental interactions, pointing to a random distribution of orthologs in the experimental data set. In contrast, the concentration of human orthologs in a network that combines both experiments and interologs is considerably diluted around the rich club and converges to *1* by definition throughout the following layers (dotted line). (c) Similarly, we determined the enrichment of proteins participating in the secretome in both the experimental and combined network of interactions in layers around the rich-club proteins. Compared to experimental interactions, we find that the addition of interologs enhances the enrichment of proteins that are secreted into the human host cell upon parasitic invasion. (d) Focusing on experimental interactions alone, we find that such links are predominately enriched in layers around the rich club, all together suggesting that the area around the rich club of proteins is predominately a matter of parasite specific activity.

Quantifying these observations, we constructed layers around the sample of nine rich-club proteins. In particular, proteins that are a certain number of steps away in the combined network of evolutionary conserved and experimental interactions constitute a new layer, allowing us to find a total of six layers. In Fig. 3a, we show the cumulative frequency distribution of the number of proteins we find running toward the outer layers, suggesting that reaching the second layer already accounts for more than half of all proteins in the network. Analyzing the composition of layers, we define enrichment as the fraction of orthologs obtained in the pool of proteins up to this layer over the total number of orthologs in the whole network compared to a random null model. If the initial placement of orthologs was random, the enrichment value would always be *1*. In Fig. 3b, we observe that orthologs seem to be randomly distributed in experimental interactions. While the concentration of human orthologs in a network that combines both experiments and interologs drops around the rich club, it ascends to *1* throughout the following layers, suggesting that the layers around the rich-club are largely unaffected by evolution and therefore constitute an area of parasite specific activity. Similarly, we determined the enrichment of proteins with an export signal in both the experimental and combined network of interactions in layers around the rich-club proteins. In particular, these proteins are the major constituents that allow the parasite to invade and gain control over the host cell by secreting them into the lumen of the human red blood cell. Compared to experimental interactions, we find that the addition of interologs enhances the enrichment of secreted proteins, confirming the particular role of the rich-club proteins for parasitic functions (Fig. 3c). Support for our conclusion that rich-club proteins arrange parasite specific functions also comes from the observation that especially in the inner layers experimentally obtained interactions dominate. Experimental observations largely cover links between parasite specific proteins [1] (Fig. 3d), suggesting that the area around the rich club of proteins not only is predominately a matter of parasite specific activity. The rich-club of proteins also appears to be the center of these activities, shaping a ‘spear-tip’ of the parasitic invasion (Detailed information about the different layers can be found in Table S2).

## Discussion

These considerable differences between experimentally determined and computationally derived interaction networks of the same species incurs interesting questions. While protein hubs in the conserved network widely are members of well known evolutionary conserved house hold functions, protein hubs in the experimental interaction set are widely found in more parasite specific features. In particular, the interesting topology revolves around such functions that are responsible for the parasites ability to invade the human host. Although such a signal might be potentially an artifact of the experimental procedures, further support for its biological significance comes from the observation that the topological vicinity around the rich club shows strong enrichment signals of parasite specific entities. This observation combined with an unprecedented topology, could putatively point to a signature of a parasitic lifestyle. Yet, the absence of any noteworthy overlaps and accordingly the rudimentary conservation of prominent cell functions [7] in *Plasmodium* remains noteworthy, especially since about 25% of proteins appear in both data sets. On the one hand, this observation might be the consequence of technical difficulties to express *Plasmodium* proteins in yeast and sampling issues that arise from choosing random fragments to screen. The latter aspect that leads to a random sample of interactions might be a reason for obtaining such a remarkable topology since random sampling methods tend to inaccurately reflect the underlying topology of an interaction network [23], [24]. On a different note, a recent assessment of protein interactions in Yeast revealed startling error rates [25]. Although such an assessment is not available for *Plasmodium* we assume that this data set is error prone as well, especially since interactions have been determined by an adapted *Y2H* approach. Therefore, the experimentally determined interaction set might carry erroneous signals. However, considering that we inferred conserved interactions predominately from Yeast, we compare experimentally obtained and evolutionary conserved interactions on a reasonably similar level of error. Alternatively, the presence of other, evolutionary conserved interactions provides computational hints that *Plasmodium* most likely contains conserved protein complexes which have been missed by the experimental approach. In the presence of a large amount of orthologous proteins in *Plasmodium* that interact in other organisms, there is no reason to assume that interactions do not occur. A reason for the absence of any evolutionary conserved interactions may also be based on limitations of sequence alignment algorithms to accurately detect homologies between eukaryotic and *Plasmodium* specific proteins. Although sharing significant similarities sequence disruptions caused by repeats and other inserts can aggravate the proper detection of evolutionary relationships of *Plasmodium* genes and proteins with other organisms [14]. Yet, the pronounced placement of orthologous proteins in interaction networks mitigates this effect since the relevant signals can still be detected even in the face of tremendous noise in interaction data as well as orthologus data [26].

Although there are considerable difficulties in the experimental and computational detection of protein interactions in *Plasmodium* that can lead to artefactual observations, we nevertheless find strong biological significance in the obtained rich-club phenomenon in the interactome of *Plasmodium*, results that potentially can point to a topological signature of a parasite specific life style. Even though results appear encouraging, the current level of the determination of the parasites interactome is in its early phases, pointing to the necessity to complete the set of interactions as well as assess their quality to further support these results.

## Supporting Information

Table S1 Protein interactions in P. falciparum. This file contains all the protein interactions in P. falciparum used in this study.
(0.84 MB XLS)

Table S2 Layers in the interactome of P. falciparum. This file contains information about the proteins in the different layers of the interactome of P. falciparum.
(0.31 MB XLS)
